# Vanadium and Manganese Carbonyls as Precursors in Electron-Induced and Thermal Deposition Processes

**DOI:** 10.3390/nano12071110

**Published:** 2022-03-28

**Authors:** Felix Jungwirth, Daniel Knez, Fabrizio Porrati, Alfons G. Schuck, Michael Huth, Harald Plank, Sven Barth

**Affiliations:** 1 Institute of Physics, Goethe University Frankfurt, Max-von-Laue-Str. 1, 60438 Frankfurt am Main, Germany; jungwirth@physik.uni-frankfurt.de (F.J.); porrati@physik.uni-frankfurt.de (F.P.); schuck@physik.uni-frankfurt.de (A.G.S.); michael.huth@physik.uni-frankfurt.de (M.H.); 2 Institute of Electron Microscopy and Nanoanalysis, Graz University of Technology, 8010 Graz, Austria; daniel.knez@felmi-zfe.at (D.K.); harald.plank@felmi-zfe.at (H.P.); 3 Christian Doppler Laboratory for Direct–Write Fabrication of 3D Nano–Probes (DEFINE), Institute of Electron Microscopy, Graz University of Technology, Steyrergasse 17, 8010 Graz, Austria

**Keywords:** FEBID, metal carbonyl, vanadium, manganese, electrical transport characteristics, CVD

## Abstract

The material composition and electrical properties of nanostructures obtained from focused electron beam-induced deposition (FEBID) using manganese and vanadium carbonyl precursors have been investigated. The composition of the FEBID deposits has been compared with thin films derived by the thermal decomposition of the same precursors in chemical vapor deposition (CVD). FEBID of V(CO)_6_ gives access to a material with a V/C ratio of 0.63–0.86, while in CVD a lower carbon content with V/C ratios of 1.1–1.3 is obtained. Microstructural characterization reveals for V-based materials derived from both deposition techniques crystallites of a cubic phase that can be associated with VC_1−x_O_x_. In addition, the electrical transport measurements of direct-write VC_1−x_O_x_ show moderate resistivity values of 0.8–1.2 × 10^3^ µΩ·cm, a negligible influence of contact resistances and signatures of a granular metal in the temperature-dependent conductivity. Mn-based deposits obtained from Mn_2_(CO)_10_ contain ~40 at% Mn for FEBID and a slightly higher metal percentage for CVD. Exclusively insulating material has been observed in FEBID deposits as deduced from electrical conductivity measurements. In addition, strong tendencies for postgrowth oxidation have to be considered.

## 1. Introduction

Focused electron beam-induced deposition (FEBID) is a very flexible direct-write nanofabrication technique representing a nanoprinting method in a scanning electron microscope (SEM). The feature size of the structures can be below ~20 nm [1], while the geometrical flexibility in 2D and 3D is exceeding the capabilities of conventional electron beam lithography approaches. The basics of the FEBID process and state-of-the-art capabilities have been reviewed in several articles [2,3,4,5]. Most importantly, fundamental properties of the deposits have been investigated, and their geometries optimized in order to prepare functional devices with the FEBID material being an active component [6,7,8,9].

FEBID depends on the electron-stimulated decomposition of volatile precursors. In this context, the most extensive body of data for precursor derivatives is focused on homometallic metal carbonyls M_y_(CO)_x_ [10,11]. The plethora of data related to the electron-induced decomposition of this class of compounds is a result of their commercial availability and typically convenient handling as well as the simplicity of the CO ligand. Nevertheless, rather surprisingly FEBID of several monometallic carbonyls has not been described in the literature to date.

Homoleptic, first-row transition metal carbonyls of groups 5–10 are known. The chemical bonding situation in metal carbonyls should be considered as a σ-donor and π-acceptor interaction. Carbonyls of V, Cr, Fe, and Ni are typically monomeric in nature, while Mn and Co derivatives are dimers. Among these compounds, all the metal carbonyls are obeying the 18-electron rule but V(CO)_6_, which is a 17-electron complex with an unpaired electron. In addition, there are also carbonyls of higher nuclearity, such as the dimer and trimers of Fe, but this reduces the volatility similar to HFeCo_3_(CO)_12_, and only few reports related to FEBID are available [12,13,14,15].

Simplistically, the decomposition of a metal carbonyl to yield a metallic deposit and gaseous carbon monoxide could be expressed as indicated in Equation (1).

(1) $$\frac{1}{n}\;M_{n}{\left(CO\right)}_{m\;\left(g\right)}\to M_{\left(s\right)}+\frac{m}{n}\;CO_{\left(g\right)}$$

However, this is the simplest dissociative process associated with carbonyl abstraction and does not consider any side reactions or potential formation of stable intermediates.

Several effects can contribute to differences in the purity of deposits. For instance, the electron-induced fragmentation is often not straightforward due to the competition of different reaction cascades. In particular, the desired electron-induced desorption of CO from the metal center can be accompanied by a cleavage of the C–O bond, which can be supported by either the electron beam-induced dissociation or the catalytic effects of the metals used. This process can result in reactive oxygen species that can either desorb or oxidize the metal and single carbon atoms that can form carbide structures or a carbonaceous matrix. The C–O bond cleavage requires ~11.2 eV [16], and even lower energies in dissociative electron attachment are described in the literature. [17] These energies are included in the energy range of the secondary electrons during FEBID. Moreover, a C–O dissociation can occur on transition metal surfaces at room temperature, which is dependent on the metal species as well as crystal facets [18]. Last but not least, the formation of stable intermediates containing carbide functionalities M_x_C_z_(CO)_y_ or carbonyls of higher nuclearity M_x_(CO)_y_ has to be considered. 

Latest results of charged particle-induced fragmentation from surface science studies suggest a partial electron-induced fragmentation of precursors in a first step, which is followed/accompanied by thermal fragmentation and CO release or the electron-induced CO cleavage leading to composites [10]. It should be noted that the highest metal contents in FEBID coincide with surface science studies showing a low-temperature thermal fragmentation of the intermediates. The thermal effects on the deposits’ composition have been demonstrated in surface science studies on partially electron-fragmented M_x_(CO)_y_ formed by electron-irradiated Ni(CO)_4_ [19], Fe(CO)_5_ [20], and HFeCo_3_(CO)_12_ [21] layers and annealing at substrate temperatures below about 60 °C. There are no comparable surface science studies on Co_2_(CO)_8_ layers, but FEBID at elevated substrate temperatures up to 60 °C can result in 80–100% Co in deposits [22]. 

Surface science studies are emulating a static process and are able to identify different fragmentation paths of the condensed precursor layer. In contrast, during FEBID contributions of (i) dominating precursor dissociation channels that can change with beam conditions or precursor density, (ii) kinetic factors influencing the completeness of precursor fragmentation, and (iii) residual gases due to lower vacuum levels in FEBID initiating side reactions will determine the materials’ composition. Moreover, it should be noted that planar FEBID deposits are typically of lower purity than pillarlike structures using the same precursor. The higher purity in the FEBID pillars is caused by the writing strategy using a static deposition spot, which is accompanied by (i) a reduced probability of interfering side reactions to occur during a deposition loop for planar deposits and (b) the potential influence of heating effects for pillar deposition [14,15]. Moreover, slightly elevated substrate temperatures can also increase the deposits’ purity when metal carbonyls are used as precursors [23]. In the context of this article, we would like to point out that FEBID techniques have been recently compared with ALD and CVD approaches for group 10 and 11 precursors [24]. Fundamentally, it is of critical importance to evaluate the decomposition of precursors at standard conditions typically used in FEBID. Hence, this study summarizes the behavior of previously neglected vanadium and manganese carbonyls in FEBID. The observed composition of the electron-induced fragmentation is supplemented with low-pressure thermal CVD results as a benchmark for exclusively thermal decomposition. 

## 2. Materials and Methods

### 2.1. Precursor Preparation/Synthesis

Sodium, Mn_2_(CO)_10_, VCl_3_, (N(C_2_H_5_)_4_)Br, methylnaphthalene, acetone, tetrahydrofurane (THF), pentane, and diethylether were purchased from Sigma-Aldrich. Diethylether, pentane, and THF were dried over sodium. All solvents including water were degassed by three freeze–pump–thaw cycles to exclude oxygen. Mn_2_(CO)_10_ was sublimed on a modified Stock apparatus at a pressure of ~1 × 10^−3^ mbar (~40–50 °C) and kept at room temperature for 4 h in the 10^−6^ mbar range using a turbomolecular pump setup prior to use as a precursor. All handling was carried out by appropriate inert gas techniques using Schlenk and Stock apparatuses. Photon-assisted degradation was avoided by covering the glass vessels used.

Vanadium carbonyl was prepared via the synthesis of (N(C_2_H_5_)_4_)(V(CO)_6_) and its conversion to V(CO)_6_ similarly to a reported procedure [25]. In a first step, a solution of VCl_3_(THF)_3_ is obtained by refluxing 10 g (64 mmol) VCl_3_ in 380 mL THF for 8 h at 339 K under argon. The VCl_3_(THF)_3_ is cooled to room temperature and added to a chilled (~213 K) solution of lithium methylnaphthalenide. The lithium methylnaphthalenide is prepared by slowly adding 54.60 g (384 mmol) methylnaphthalene to a suspension of 1.77 g Li (256 mmol) in 180 mL THF over the course of 30 min and stirring at ~298 K for 8 h. The combined mixture of VCl_3_(THF)_3_ and lithium methylnaphthalenide is stirred for 12 h and allowed to warm to ~263 K. Subsequently, the reaction mixture is stirred vigorously, and the atmosphere swapped to pure CO (*Caution: CO is a poisonous gas and should only be handled with proper precautions!)* and kept for 24 h at room temperature while replacing the consumed CO. The black solution is filtered, and the residue is washed with 20 mL THF. The filtrate volume is reduced to ~50 mL, and 500 mL pentane is added to precipitate the crude lithium salt as dark brown tar, which is washed several times with pentane to remove methylnaphthalene. The residue is dried in vacuo, redissolved in 300 mL acetone, and added to a solution of 16.14 g (77 mmol) (N(C_2_H_5_)_4_)Br in 200 mL ethanol. The crude product is precipitated by reducing the volume to ~50 mL and adding 600 mL water. Water is removed by filtration, the product redissolved in 300 mL THF and precipitated as yellow solid after reduction of the volume to ~30 mL and addition of 20 mL diethylether. 

In a second step, vanadium carbonyl is obtained by adding 5 g (N(C_2_H_5_)_4_)(V(CO)_6_) to 40 mL of phosphoric acid (dried by the addition of ~5 g of phosphorous pentoxide), leading to the formation of the vanadium carbonyl hydride, which readily decomposes to V(CO)_6_. The crude product is collected in a specially designed Schlenk tube with the stopcock attached to the lower end of the tube by applying a dynamic vacuum. A complete removal of water is achieved by adding phosphorus pentoxide to the crude product. Finally, pure vanadium carbonyl is obtained by low-temperature resublimation (~1 × 10^−3^ mbar on a modified Stock apparatus), keeping the crude product at 298 K and collecting the V(CO)_6_ in a cooling trap kept at 77 K.

The precursor is sensitive to photodegradation, which is enhanced by reducing the overall pressure in the vessel. Nevertheless, avoiding direct exposure to light, V(CO)_6_ can be stored for several weeks under Ar and in evacuated stainless steel containers at 253 K.

### 2.2. FEBID Process

FEBID was performed using a dual-beam SEM/FIB (FEI, Nova NanoLab 600, Thermo Fisher Scientific, Waltham, MA, USA) equipped with a Schottky electron emitter. The precursors were injected into the SEM chamber using a custom-built gas injection system (GIS) via a capillary with an inner diameter of 0.5 mm. Typically, the capillary was positioned 100 µm laterally and vertically shifted from the intended deposition spot on the substrate at a substrate–capillary angle of 15°. The substrates used in the study are either (i) (0001)-oriented sapphire single crystals coated with a 250 nm Au film with an 8 nm Cr adhesion layer or (ii) p-doped (100) Si with a 300 nm SiO_2_ coating and predefined Au microelectrodes. After the substrate was mounted in the microscope, air plasma cleaning was always performed to reduce the hydrocarbon level in the chamber. Prior to deposition experiments the system was pumped for at least 24 h. The residual water content was reduced by using a Meissner trap for 4 h. This procedure resulted in a background pressure of <3.6 × 10^−7^ mbar in the microscope. The precursor container was kept at 293 K (Mn) for the deposition to retain the vapor pressure at an acceptable level. The total pressure within the SEM chamber during the deposition was typically kept below 2 × 10^−6^ mbar and was regulated via a needle valve. For storage, the V(CO)_6_ was kept below 263 K to prevent thermal degradation of the precursor and allowed for reaching the deposition temperature of 278 K 2 h prior to use.

FEBID process parameters, including electron beam current and voltage, were varied to study the effects on the deposits’ properties. The pitch (20 nm in x- and y-direction) between deposition events and the dwell time (1 µs) were kept constant. The deposits for two-point transport measurements were 4.0–5.2 µm × 1 µm with a thickness/height in the range of 100–180 nm. FEBID samples for four-point characterization had typical dimensions of 10 µm × 1 µm with a thickness of ~100 nm for V-based material and ~200 nm for Mn-based FEBID material. 

CVD has been carried out in a home-built cold-wall reactor using high-frequency heating of a graphite susceptor for indirect heating of sapphire (0001) and Si (911) substrates. The substrates are attached to the graphite susceptor by silver paste to ensure efficient thermal contact. Substrate temperatures have been limited to 473–673 K. The metal carbonyls were introduced in the reactor through a glass flange applying dynamic vacuum (~10^−6^ mbar) while keeping the precursor temperatures in the range of 263–283 K. Typically, 30–40 mg of the carbonyls were used as source for the growth experiments, and the growth was carried out for 60–120 min. A similar CVD setup has been described in the literature for the growth of thin films and nanostructures using molecular sources [26,27].

### 2.3. Transport Measurements and Compositional Analysis

Au microelectrodes for electrical characterization were prepared by ultraviolet contact photolithography and sputtering of an 8 nm Cr adhesion layer, followed by 75 nm Au on SiO_2_ (300 nm)/p-Si substrates (CrysTec GmbH, Berlin, Germany). Four-point electrical transport measurements were carried out in the temperature range of 2–300 K in a variable-temperature insert mounted in a ^4^He cryostat. In order to identify the conditions of lowest material resistivity while keeping a defined deposition pattern for the variable temperature experiments, in situ two-point electrical transport measurements were carried out inside the SEM after FEBID [28]. Standard measurements were performed using a Keithley SourceMeter 2400 (Solon, OH, USA) and an Agilent 34420A nanovoltmeter (Santa Clara, CA, USA). 

Lamellae for cross-sectional TEM of the deposits were prepared via a standard focused ion beam (FIB) milling procedure utilizing Ga ions and MeCpPtMe_3_ as a precursor in a FIB/SEM dual-beam microscope, FEI NOVA 200 (Thermo Fisher Scientific, Waltham, MA, USA). The lift-out and initial milling step were carried out with an acceleration voltage of 30 kV, and the final milling step was performed at 5 kV. The resulting lamella was mounted onto an OmniProbe copper-based lift-out grid and directly transferred to the TEM. TEM observations were carried out on a probe-corrected FEI Titan^3^ G2 microscope operated at 300 kV in scanning mode. The microscope is equipped with a Gatan Quantum imaging filter for electron energy loss spectrometry (EELS) and a high-sensitivity four-quadrant SDD (Super-X) detector for energy dispersive X-ray analysis (EDX). Data acquisition and analysis was performed using the Gatan Microscopy Suite (version 3.4; Gatan, Inc., Pleasanton, CA, USA) and Velox (version 3.0) by Thermo Scientific (Waltham, MA, USA). 

The topographical features of the deposits were determined by atomic force microscopy (AFM) operated in tapping mode (nanosurf (Liestal, Switzerland), easyscan2). After the FEBID growth, the sample composition was deduced from energy dispersive X-ray analysis (EDX) at a beam energy of 5 kV for Mn-based material and 11 keV for V-based FEBID material. Note that deposits characterized by EDX were thick enough to avoid prominent contributions from the Au substrate layer. Error bars represent variations between several EDX spectra recorded for deposits using a defined set of parameters. In addition, the standardless quantification carried out in this study provides an estimate of the actual composition, and thus, it is not as accurate as EDX using defined material compositions for calibration. A slight overestimation of the carbon content could be caused by additional deposition during EDX associated with residual carbon sources in the background gas. These potential minor systematic errors do not change any tendencies discussed in the paper, especially because C deposition during EDX on the Au substrate is negligible. Due to the overlap of the V_L_ (0.511 keV) and the O_Kα_ (0.523 keV) signal, the actual oxygen content was not determined. For comparison of the V/C ratio, the V_L_ peak was used as the accurate V content in relation to the C_K_ signal. A meaningful quantification for specific cases such as the V/O pair is not trivial and cannot be provided by standardless automated EDX microanalysis [29]. The challenges might be reduced by using the newest detector hardware and simulation software packages as demonstrated for similarly challenging samples as PbS [30]. However, to the best of our knowledge no data on such a study for V/O are reported in the literature. 

For characterization of CVD thin films by X-ray diffraction, a Bruker D8 Discover (Billerica, MA, USA) was used in a Bragg–Bretano geometry. Match! software (crystal impact, Bonn, Germany) was used for data analysis. 

## 3. Results and Discussion

Freshly sublimed V(CO)_6_ and Mn_2_(CO)_10_ are stable under the given conditions and can be stored in stainless steel containers or glass vessels, which are attached to the GIS of the SEM or the CVD reactor. 

### 3.1. Vanadium Carbonyl Precursor

**Figure 1:**
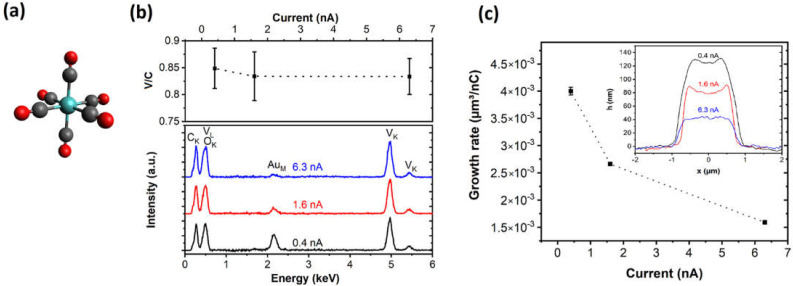
(**a**) Schematic illustration of the monomeric V(CO)_6_ precursor used in this study. (**b**) The V/C ratio of the deposits is determined by EDX, and the variation is shown in response to changes in the beam current used at a constant acceleration voltage of 5 kV, including the corresponding EDX data. Further FEBID parameters include a deposition area of 1.4 µm × 1.4 µm a 20 nm pitch in the x- and y-direction, and a dwell time of 1 µs. The substrates for the EDX studies are 250 nm Au layers with Cr (8 nm) adhesion layer on sapphire (0001) single crystals. (**c**) The inset shows height profiles of V-based FEBID material with a nominal 1.4 µm × 1.4 µm area deposited at a 5 kV beam voltage for different beam currents (as indicated) but identical doses of 40 nC·µm^−2^. Further deposition parameters include: a pitch of 20 nm in the x- and y-direction and a dwell time of 1 µs. The volume growth rate per dose is determined using the deposit volumes and is plotted in relation to the beam current used for FEBID.

The solid black V(CO)_6_ precursor is a monometallic molecule with octahedral coordination of the V metal center, as illustrated in Figure 1a. The small intermolecular interactions lead to a high volatility of this compound. The degree of electron-induced dissociation during the writing procedure is studied by altering the deposition parameters, such as beam current and voltage and changes in the precursor supply, while keeping the dwell time and pitch constant. Except for the pressure-related decomposition series, all FEBID experiments have been conducted at overall chamber pressures of 0.85 × 10^−6^–1.03 × 10^−6^ mbar.

Figure 1b illustrates the constant V/C (~0.85) ratio in the FEBID deposit when currents in the range of 0.4–6.3 nA are used at a 5 kV beam voltage. A higher acceleration voltage of 11 kV is necessary for the EDX investigations in order to determine the V content using the V_K_ peak. The low-energy part of the spectrum is not suitable for this analysis due to the overlap of the V_L_ and the O_K_ signals. Corresponding examples of EDX spectra obtained under the different beam conditions are also included in Figure 1b. Similarly, the precursor flux, which is implicitly recorded as the chamber pressure at 0.5–1.8 × 10^−6^ mbar with a background pressure of 3.8 × 10^−7^ mbar, has a minor effect on the V/C ratio remaining in the window of ~0.73–0.83 (Figure S1 of the SI). Increasing the beam voltage leads to a drop in the V/C ratio to ~0.67 at 20 kV, which could be caused by a change in the deposition regime associated with dominating fragmentation channels (Figure S2). The generally observed oxygen signal in the overlapping low-energy part of the spectrum still allows for the general conclusion that for all the FEBID deposits, this contribution is not altered by large amounts with respect to the C_K_ and V_K_ contributions. 

AFM images show only a minor influence of surface diffusion on the deposits’ shape evolution with the edge region being slightly higher than the center (Figure 1c, inset). The observation can be assigned to diffusion effects encountered in the serpentine deposition pattern used in this study [31,32]. The deposition is considered to occur in the diffusion-enhanced regime as detailed in the literature and indicated by this sort of edge effect [33,34]. It should be noted that this edge enhancement is not observed for the structures used in the following electrical characterization due to the different loop times associated with the different geometry. The volume determined from the AFM analyses is used to determine the growth rate. Figure 1c shows the volume-based material growth rate per electrical charge, which decreases with an increasing beam current. A similar trend has been described for cobalt silicide [34] and PtC_x_ deposition [32] and is indicative of a precursor-limited regime since the growth rate is decreasing with an increasing beam current. 

**Figure 2:**
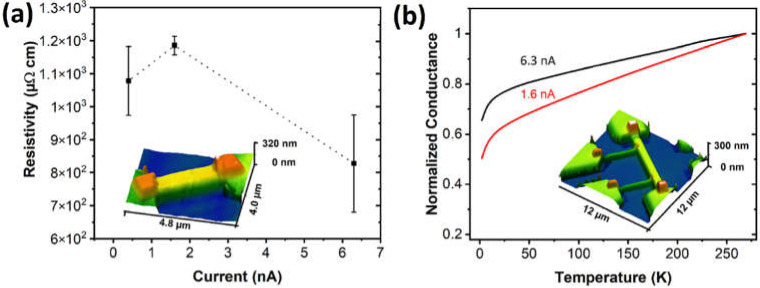
(**a**) Resistivity vs. electron beam current used for FEBID recorded in the SEM chamber after the material growth. The devices are investigated in a two-point configuration, as illustrated in the AFM image of the FEBID deposit bridging Au microelectrodes. (**b**) Temperature dependence of the conductance normalized at 285 K for V-based FEBID samples prepared at 1.6 nA and 6.3 nA at 5 kV, respectively. The AFM image in the inset shows a typical deposit used for these studies based on four-terminal devices.

The electrical resistivity determined under constant voltage bias conditions of the V-based FEBID deposits shows only minor changes when the beam current for the deposition is altered in the range of 0.4–6.3 nA at 5 kV, resulting in resistivities of 0.8–1.2 × 10^3^ µΩ·cm with the lowest value obtained for the highest beam current (Figure 2a). The values are recorded in two-terminal devices and as such are not corrected for contact and lead resistance contributions. However, complementary four-probe measurements indicate that these parasitic contributions are negligible for the V-based FEBID material written between Au microelectrode contacts. Note that the postgrowth electron irradiation curing at a 5 kV beam voltage leads to an increase in resistance of the deposits (Figure S3) and not to the typically observed reduction in resistance as described in the literature for different material systems [34,35,36,37]. A speculative explanation of the observed effect is oxidation due to reaction with residual water in the chamber during the electron curing process. However, no compositional variation was detected in EDX analysis. The FEBID system used in this study is optimized for low concentration of background gas pressures, and therefore, this speculation has not been validated.

In this context, it should be mentioned that vanadium oxides show interesting and desirable physical properties [38]. Therefore, it might be an opportunity to evaluate whether VO_x_ can be prepared by electron-supported oxidation using water. The electron-stimulated oxidation by feeding dry oxygen gas via a GIS during the electron curing process at 6.3 nA and 5 kV at a total chamber pressure of ~2 × 10^−6^ mbar was ineffective. The electronic properties and the overall composition remained unaltered in such experiments when compared with the postgrowth electron irradiation in the absence of oxygen.

A typical four-terminal device is shown in the AFM image of Figure 2b and has been used to determine the temperature-dependent electrical conductivity of the as-prepared V-based FEBID nanostructures. The graph in Figure 2b illustrates the changes in conductance normalized to the respective room temperature values. The V-based FEBID deposits written at 1.6 nA and 6.3 nA at 5 kV can be considered as being on the quasimetallic side of the insulator–metal transition [35]. A similar temperature dependence of the electrical conductance has been described for several FEBID-derived material systems [15,39].

**Figure 3:**
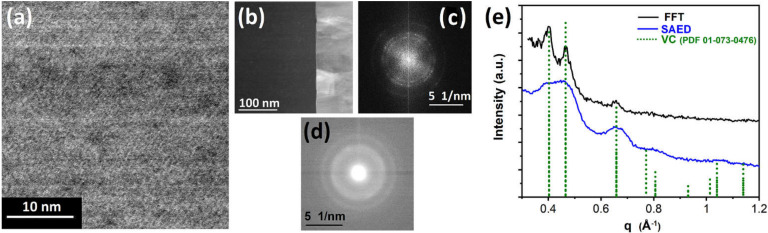
(**a**) High-resolution bright-field image of the V-based FEBID material shows phase/diffraction contrast suggesting particle sizes in the range of 2–5 nm. The high-resolution image is part of a lamella prepared from a ~200 nm thick FEBID deposit written on top of a gold layer, as illustrated in (**b**) with the brighter Au layer in this high-angle annular dark-field image (HAADF). (**c**) The fast Fourier transform image using the TEM image in (**a**) is reduced to the central, significant part illustrating specific ring structures, which can be supported by (**d**) nanobeam electron diffraction images providing information on the crystalline fraction of the FEBID material. (**e**) The graphic representation of information as a rotational profile that can be extracted from (**b**,**d**) allows the assignment of a crystal phase, such as VC, which can be also described as a generalized solid-solution VC_1−x_O_x_.

TEM investigations were performed to provide information on the deposits’ microstructure. In order to avoid postgrowth modification, the as-grown V-based FEBID material written at 6.3 nA (5 kV) was covered with a PtC_x_-FEBID layer and subsequently with a PtC_x_-FIBID coating using MeCpPtMe_3_ as a precursor. Figure 3a shows a high-resolution STEM image of the V-based FEBID material. The phase/diffraction contrast in the bright-field micrograph suggests crystalline particle sizes of 2–5 nm located in an amorphous matrix. The FEBID material is grown on top of the microcrystalline gold layer, which is illustrated in the high-angle annular dark-field (HAADF) image in Figure 3b. A fast Fourier transformation (FFT) image generated from Figure 3a reveals the typical ring structure that is expected for a polycrystalline material. Figure 3c shows the magnified area of interest in the FFT, which allows for determining the lattice plane spacings and subsequently identifying the crystalline phase of the nanoparticles similar to the nanobeam electron diffraction (NBED) of a similar area in Figure 3d. The locations of the rings in the FFT and NBED are better visible from the corresponding rotational averaged profiles provided in Figure 3e. Accordingly, the diffraction distances can be attributed to the reflections originating from a cubic crystal phase, which can be either the isostructural VC, VO, or a solid solution VC_1−x_O_x_. Hence, the reference of the cubic VC phase in Figure 3e is a representative for all the aforementioned compositions of the possible crystalline material.

**Figure 4:**
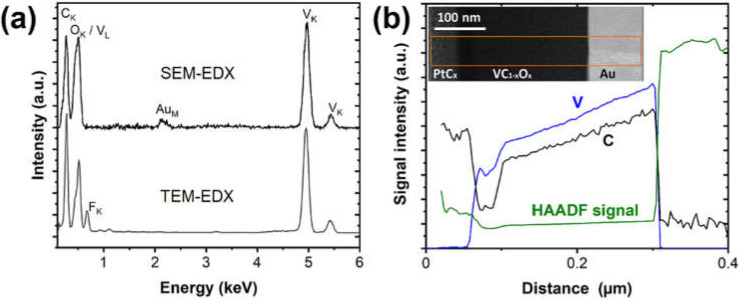
(**a**) Comparison of EDX spectra obtained in the SEM and TEM equipment used for analysis. F in the TEM sample is a residue of the lamella preparation and often observed in the samples. (**b**) EELS line scans for C and V_L_ show a similar trace with increasing thickness of the TEM lamella towards the Au substrate material. The scan is carried out along the line indicated in the HAADF image with contributions of a PtC_x_ FEBID protection layer, FEBID VC_1−x_O_x_, and the Au substrate.

Analysis of the EDX spectra obtained from the FEBID bulk of the TEM lamella shows a distinct difference when compared with the spectra recorded in the SEM chamber as described before for V-based FEBID material deposited at 6.3 nA and 5 kV. The most significant difference in Figure 4a is a reduced signal of the peak area associated with the overlapping V_L_ and O_K_ signal when compared with the V_L_ signal. This indicates that the oxygen content should be slightly lower within the deposit. The most likely explanation is a postgrowth alteration of the materials’ composition during the EDX investigation for these materials. The remaining water in the chamber could lead to an alteration of the material composition due to oxidation as already suggested (vide infra). In this process, the carbon content could be slightly reduced, and at the same time, the oxophilic metal is oxidized. An indication for the suggested process is a top layer in between the FEBID PtC_x_ and the VC_1−x_O_x_, as illustrated in Figure 4b. This layer of ~25 nm shows a higher content of low Z elements in the dark-field TEM image visible as a brightness trace of the HAADF signal in Figure 4b. Moreover, the TEM-EDX illustrates a larger peak area of the region associated with the V_L_ and O_K_ signal similar to the SEM-EDX (Figure S4) at the interface region between a V-based deposit and a PtC_x_ layer. Thus, an electron-induced oxidation after the growth of the original VC_1−x_O_x_ in the SEM due to residual water can be assumed. 

The electron energy-loss spectroscopy (EELS) line scan along the indicated area of the HAADF image in Figure 4b illustrates a constant composition/ratio of V/C. The increasing signal intensity with the same slope is caused by the wedge shape of the TEM lamella. Transitions between compositional variations are also visible in the HAADF image showing the different materials containing FEBID PtC_x_ as a protective layer, a surface VC_1-y_O_y_ layer, the bulk VC_1−x_O_x_ FEBID, and a Au substrate layer. EELS analysis is suffering from similar limitations in the compositional analysis for the vanadium and oxygen signals. Therefore, no oxygen quantification by EELS is provided herein. Nonetheless, a constant V/C ratio in the V-based FEBID material can be deduced.

**Figure 5:**
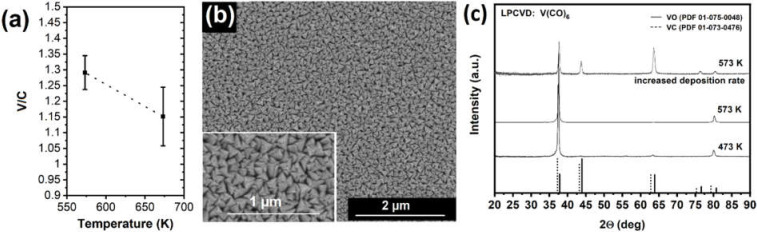
(**a**) Composition of CVD coatings determined by EDX for films prepared at different temperatures using V(CO)_6_ as a precursor. The elemental V/C ratio is in all cases above 1. (**b**) SEM image shows a homogenous V-based CVD coating deposited at 573 K on Si (911). The inset shows grains with pronounced faceted morphologies on the surface of a film, which is grown at low precursor temperatures of 263 K. (**c**) XRD pattern recorded at room temperature for films grown at substrate temperatures ranging from 473 to 573 K showing preferred orientation. Increased deposition rate leads to deposits with a lower degree of orientation and crystal sizes, allowing an unequivocal identification of a cubic crystal phase corresponding to VC_1−x_O_x_.

To the best of our knowledge, there is no report in the literature on the thermal decomposition of V(CO)_6_. Therefore, temperature-induced decomposition has been investigated in a low-pressure cold-wall CVD reactor. When compared with the FEBID material, the low-pressure CVD shows a higher V/C ratio of 1.1–1.3 (FEBID: 0.63–0.85) for substrate temperatures of 473–673 K, as illustrated in Figure 5a. This discrepancy is not attributed to an additional decomposition of hydrocarbons in the residual gas during EDX analysis due to negligible deposition in control experiments on pristine substrates, but rather to a different decomposition path/mechanism.

The low-pressure CVD process using V(CO)_6_ results in thin films of a dark bronze color, which have been deposited on Si (911) and sapphire (0001) single crystals. The composition has been obtained from at least three different films prepared with identical parameters. Figure 5b shows a typical SEM image suggesting columnar growth with well-defined facets. The phase identification is easier for less oriented growth, which is observed for higher growth rates, keeping the V(CO)_6_ precursor reservoir at 273 K (Figure 5c). In accordance with references for VC and VO, a cubic crystal symmetry belonging to the space group Fm-3m can be assigned. The reflections are located between both reference patterns, which suggests either highly substoichiometric VC_1−x_ or a solid solution of VC_1−x_O_x_ due to the isostructural nature of both cubic phases. It should be noted that the slower growth rate when the precursor is kept at 263 K does not lead to a different deposit composition but an oriented growth along the 〈111〉 axis, thus reducing the XRD pattern to dominating reflections at 2Θ values of 37.5° and 80.0°. The cubic VC_1−x_O_x_ phase is also observed in the FEBID samples, as described in Figure 3e. 

The crystal phase observed in FEBID and low-pressure CVD using V(CO)_6_ match well, while the V/C ratio differs with a higher metal/carbon ratio for CVD material with values up to 1.3. Hence, the purely thermal decomposition at elevated temperatures of 573–673 K provides materials with lower carbon content, thus suggesting a slightly more efficient thermal CO cleavage. In conclusion, V(CO)_6_ does not provide access to metallic V but might be used for studies targeting vanadium oxides since the precursor is not susceptible to hydrolysis, but oxidation by molecular oxygen has to be prevented. 

### 3.2. Manganese Carbonyl Precursor

**Figure 6:**
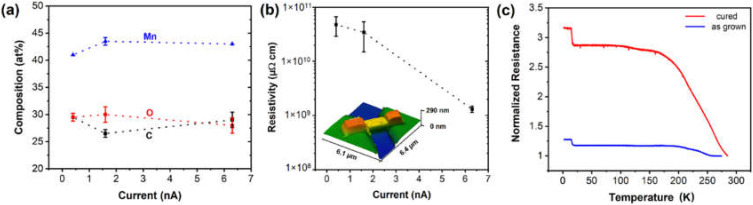
(**a**) EDX analyses show the composition of FEBID materials deposited at a 5 kV beam voltage and in response to the electron current in the range of 0.4–6.3 nA. (**b**) Electrical resistivity of the Mn-based FEBID material prepared at 0.4–6.3 nA and 5 kV. The values were derived from the resistance measurements of as-prepared material in the SEM by using the two-terminal device geometry and their geometrical factors determined by AFM. (**c**) Temperature-dependent electrical resistance evolution of two-terminal devices prepared at beam parameters of 6.3 nA and 5 kV and curing at 6.3 nA (1000 C/µm^2^).

The freshly resublimed Mn_2_(CO)_10_ was used for FEBID experiments with the precursor reservoir remaining at 293 K. Mn_2_(CO)_10_ is a dimer, and therefore, the volatility is lower than for the monomeric metal carbonyls. Changes in the FEBID deposition parameters were limited to the beam current and voltage, while keeping the dwell time and pitch constant. Figure 6a illustrates the composition of the FEBID material analyzed using EDX measurements. The Mn content is constant at ~43 at% for current and voltage variations with only the smallest currents of 0.4 nA showing a slightly lower Mn content. The C/O ratio reflects a 1:1 composition and represents one remaining CO fragment per 1.5 Mn atoms. Thus, the FEBID deposits’ composition can be expressed as MnC_0.65_O_0.65_ with only minor variation upon changes in beam current. Increasing the beam voltage shows a tendency of decreasing C content and C/O ratios of 0.65, which could hint towards increasing impact of residual water, leading to a removal of carbon in the deposits, while the M/O ratio essentially retains values of ~1.5 (Figure S5). Growth rates observed show moderate growth in the range of 1.8 × 10^−3^–0.4 × 10^−3^ µm^3^·nC^−1^ for FEBID deposition at 0.4–6.3 nA and a 5 kV beam voltage.

The electrical resistivity of MnC_0.65_O_0.65_ FEBID deposits was investigated in a two-point geometry, while neglecting the contact and lead resistances (Figure 6b). In general, very high resistivity values of 0.15–5 × 10^10^ µΩ·cm were observed for deposits using beam current values of 0.4–6.3 nA at a 5 kV beam voltage. Similar to other FEBID deposits, postgrowth irradiation of material prepared using 6.3 nA at 5 kV with the same beam conditions reduces the resistivity by one order of magnitude, but the overall values remain very high >1 × 10^8^ µΩ·cm (Figure S6). Four-probe electrical measurements reveal a minor effect of contact and lead resistances with the aforementioned insulating properties dominating the deposits’ electrical properties. 

Even though the FEBID material is mostly insulating, the variable-temperature electrical transport measurements show signatures of a phase transition (Figure 6c). The low-temperature sharp transition occurs with an abrupt onset at ~15 K. To the best of our knowledge, there is no specific oxidic or metallic Mn-based phase described in the literature with a transition temperature close to this value. Moreover, the nonequilibrium nature of crystallization of the oxides at ambient temperatures leads to different metastable oxide phases, and as a result, multivalence mixtures of manganese oxides are often obtained [40]. This general complexity accompanied with a well-known size effect for transition temperatures [41] allows only a qualitative assessment of the observed effect. In the temperature regime below 15 K, the resistance remains unaltered, and similarly negligible variations can be observed between 25 and 170 K, while the resistance decreases at temperatures above 190 K up to room temperature. In general, the microstructural investigation is very challenging, and a slight variation in the electrical properties is already observed during the transport between two measurement setups due to the oxidation tendency of the material. Hence, at this stage no specific microstructural characterization has been carried out. 

In addition, strong variations in oxygen content were observed when no Meisner trap was used before FEBID. Control experiments without prior water removal show a significantly higher oxygen content and a simultaneous reduction in carbon content to yield MnC_0.19_O_0.90_ FEBID deposits instead of the MnC_0.65_O_0.65_ composition described before. This result points toward the possibility of electron-induced oxidation by residual water. Hence, studies under a controlled water atmosphere to yield defined manganese oxide deposits could be envisioned. The wealth of various physical phenomena observed in the different manganese oxides could justify similar experiments as carried out for the purification of Pt, Pd, and Au in order to remove C [42,43,44,45].

**Figure 7:**
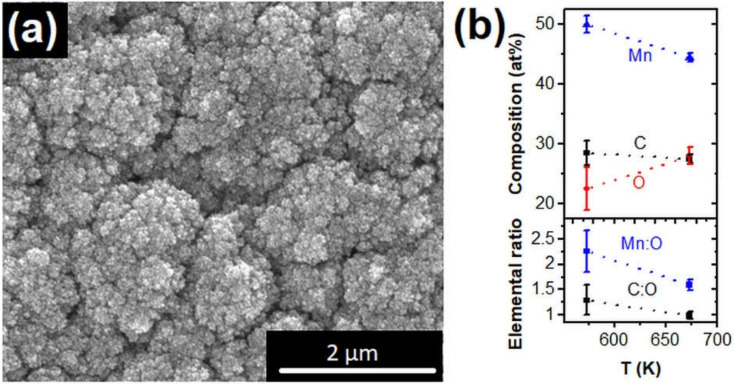
(**a**) SEM image of a CVD film deposited at 673 K using Mn_2_(CO)_10_ as precursor. (**b**) illustrates the composition of Mn-based CVD deposits determined by EDX analysis.

Thermal decomposition of Mn_2_(CO)_10_ by low-pressure CVD on sapphire (0001) substrates leads to the formation of thin films with metallic appearance. The XRD analysis reveals predominantly the cubic MnO phase and often other nonidentified reflections, which are typically changing during the measurement due to ongoing oxidation when being exposed to ambient conditions. The sensitivity to oxidation and delamination of the films after a few days at ambient conditions can be traced back to the porosity of the nanostructured thin Mn-based film with high surface roughness. Figure 7a shows a SEM image of a Mn-based film prepared at substrate temperatures of 673 K. Similar to the aforementioned FEBID results, the EDX analysis of as-synthesized CVD layers at temperatures between 573 and 673 K shows the presence of carbon (~27–28 at%), oxygen (~22–27 at%), and 45–50 at% Mn (Figure 7b). This is in line with earlier reports on the thermal decomposition of Mn_2_(CO)_10_ for deposition temperatures of ~623 K, indicating in situ oxide formation and also signatures of carbide in X-ray photoelectron spectroscopy [46]. Thermal C–O bond dissociation is known to occur on transition metal surfaces. However, this is usually a minor reaction channel in the thermal decomposition of most metal carbonyls. Therefore, these results are an indication that more pronounced thermal effects in FEBID using Mn_2_(CO)_10_ will not lead to significantly higher Mn content under the given experimental conditions/vacuum levels. In contrast to the described V-based material, the formation of lager amounts of carbide could be excluded for the Mn-based deposits; however, the strong tendency towards oxidation and the existence of multiple metastable phases/compositions make the investigation of Mn_2_(CO)_10_-derived FEBID and CVD deposits very challenging.

### 3.3. Carbonyl Precursors in FEBID

The large body of data on different homoleptic metal carbonyls allows us to draw overarching conclusions on the most important class of FEBID precursors. The partial release of CO by (CO)_x_M-CO fragmentation upon electron irradiation is a common feature of all metal carbonyls as suggested by gas-phase and surface science studies [10]. In general, it should be noted for the expected compositions that the carbon/oxygen ratio should be ≥1 considering potential thermal or electron-induced oxygen loss after C–O cleavage by metal–oxo species, while ratios <1 are indicative of additional reactions with residual water in the vacuum chamber. Similar to the thermal fragmentation of precursors in CVD, it should be emphasized that electron dissociation of any molecule in FEBID are statistical processes. Therefore, the overall fragmentation in FEBID can be dependent on the experimental conditions, which may include changes in the dominating dissociation path and thus the composition. 

**Figure 8:**
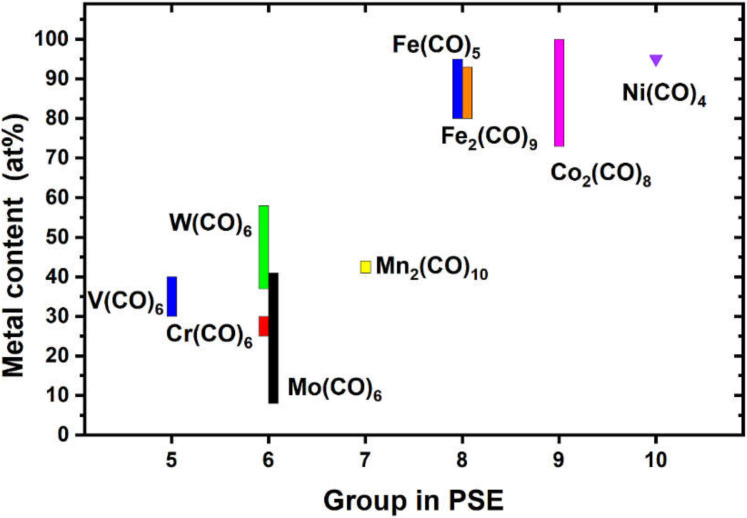
The maximum metal contents for FEBID deposits derived from homometallic carbonyls were deduced from the literature [12,13,23,47,48,49,50,51,52,53,54,55,56,57,58] and complemented by the results in this study. The V metal content is considered based on approximations of the oxygen content.

Figure 8 summarizes the maximum metal contents reported for the different homometallic, homoleptic metal carbonyl precursors tested for FEBID to date [12,13,23,47,48,49,50,51,52,53,54,55,56,57,58]. The graphic reveals two clusters of FEBID materials. The carbonyls of groups 8–10 (iron triad) are reported to produce metal content over 80 at% in FEBID deposits, while carbonyls of groups 5–7 typically contain less than 50 at% metal in the FEBID material. The investigated V and Mn carbonyl precursors here produce FEBID materials with an intermediate metal content of ~30–45 at%.

To the best of our knowledge, besides the V(CO)_6_ investigated in this study, only W(CO)_6_ has been described to form carbide species in FEBID. However, the allocation of carbide formation has been mostly supported by the transport characteristics, since WC_x_ is an interstitial metal carbide with variable carbon content. 

A significant number of FEBID-derived W-based materials can be considered as WCO_0.7_ when W(CO)_6_ is used as a precursor, and higher beam currents (>~3.6 nA) are applied for the deposition [23,28,39,59]. The formation of a carbide fraction in the W-based deposits can be related to positive metal ions that can form through electron–molecule interactions via dissociative ionization, where a significant number of carbide-type WC^+^ and WC(CO)^+^ ions are observed in gas-phase studies [60]. The microstructural TEM investigations on V-based FEBID material derived from V(CO)_6_ reveal that nanoparticles owning a cubic phase are present, which matches very well to the VC reference, and the oxygen content appears to be well below the atomic percentages of V and C. Even though there is no dominating fragmentation channel with carbide fragments associated with gas-phase studies on electron-stimulated fragmentation of V(CO)_6_ [61], the presented results here suggest the efficient formation of vanadium (oxy)carbide species in FEBID. 

FEBID using Mn_2_(CO)_10_ results in an insulating MnC_0.65_O_0.65_ material, which also oxidizes quickly. The metal content of ~43 at% is close to the maximum Mn content in thermal CVD under similar vacuum conditions, which illustrates that this is the highest purity that can be obtained by either FEBID or thermal decomposition under these conditions. Surface science studies in ultra-high-vacuum conditions might allow the determination of a higher metal content, but under FEBID conditions, a much higher manganese content is most likely prevented by either incomplete CO abstraction or additional oxidation by the residual water in the SEM chamber. 

## 4. Conclusions

The compositions of materials derived by FEBID and thermally induced CVD are similar for both V(CO)_6_ and Mn_2_(CO)_10_. In general, changes in deposition current and voltage do not significantly affect the material composition in the electron-induced deposition. 

FEBID using V(CO)_6_ results in VC_1−x_ O_x_ material containing V/C ratios of ~0.63–0.86 with electrical resistivities in the range of 0.8–1.2 × 10^−3^ µΩ·cm. EDX analysis in the TEM suggest that the oxygen content might be overestimated in the SEM/EDX analysis, which might be caused by surface oxidation during imaging or processing, such as PtC_x_ layer deposition. The temperature-dependent normalized electrical conductance analysis is indicative of a material on the quasimetallic side of the metal to insulator transition. In addition, the crystalline fractions in the microstructural characterization of V-based FEBID material reveal a cubic VC_1−x_O_x_ phase in agreement with the crystalline phase derived by low-pressure CVD. The thermal fragmentation in CVD yields higher V/C ratios of 1.1–1.3, indicating that the dominating fragmentation path in the FEBID experiments presented here favor C incorporation. 

In contrast, FEBID using Mn_2_(CO)_10_ resulted in an electrically insulating deposit containing Mn in the range of ~40 at%, which is very similar to the composition of CVD-derived thin films. However, the materials are highly receptive to oxidation and ~0.67 CO ligands remaining in the FEBID deposit according to the EDX data. This suggests that higher metal contents could potentially be obtained under UHV conditions and potentially even slower growth rates.

## Data Availability

Data is contained within the article or Supplementary Material.

## Supplementary Materials

The following are available online at https://www.mdpi.com/article/10.3390/nano12071110/s1. Figure S1: Pressure-dependent V/C ratio in FEBID; Figure S2: V/C ratio in FEBID materials with voltage changes; Figure S3: Effect of postgrowth electron curing on resistivity in V-based FEBID material; Figure S4: Comparison of EDX spectra from TEM and SEM on V-based deposits; Figure S5: Composition of Mn-based FEBID material upon changes, in beam voltage; Figure S6: Effect of postgrowth electron curing on resistivity in Mn-based FEBID material.
