# Supplementary material for: Vanadium and Manganese Carbonyls as Precursors in Electron-Induced and Thermal Deposition Processes
Source: Nanomaterials (Basel). 2022 Mar 28;12(7):1110. doi: 10.3390/nano12071110 (PMC9000455; doi:10.3390/nano12071110)
Supplement: Supplementary file 1 [file nanomaterials-12-01110-s001.zip › nanomaterials-1638524-supplementary.pdf]

nanomaterials

Supporting Information

Vanadium and Manganese Carbonyls as Precursors in Electron-Induced and Thermal Deposition Processes

Felix Jungwirth [1], Daniel Knez [2], Fabrizio Porrati [1], Alfons G. Schuck [1], Michael Huth [1], Harald Plank [2,3] and Sven Barth [1,*]

[1] Institute of Physics, Goethe University Frankfurt, Max-von-Laue-Str. 1, 60438 Frankfurt am Main, Germany; jungwirth@physik.uni-frankfurt.de (F.J.); porrati@physik.uni-frankfurt.de (F.P.); schuck@physik.uni-frankfurt.de (A.G.S.); michael.huth@physik.uni-frankfurt.de (M.H.)
[2] Institute of Electron Microscopy and Nanoanalysis, Graz University of Technology, 8010 Graz, Austria; daniel.knez@felmi-zfe.at (D.K.); harald.plank@felmi-zfe.at (H.P.)
[3] Christian Doppler Laboratory for Direct–Write Fabrication of 3D Nano–Probes (DEFINE), Institute of Electron Microscopy, Graz University of Technology, Steyrergasse 17, 8010 Graz, Austria
* Correspondence: barth@physik.uni-frankfurt.de; Tel.: +49-69-7984-7261

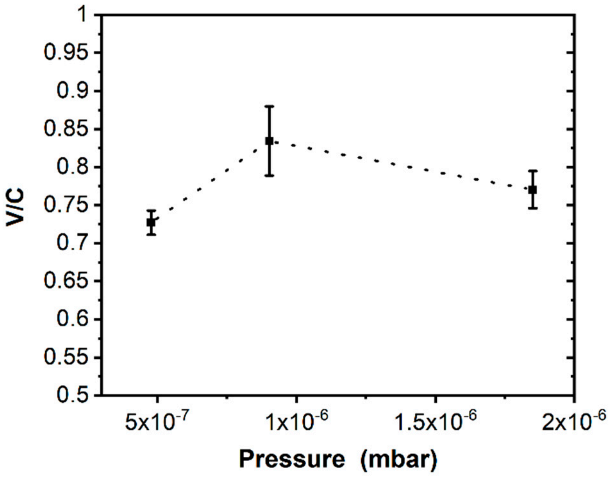

Figure S1. Evaluation of variation in V/C ratio using $V(CO)_6$ precursor in FEBID upon changing the precursor pressure. The deposit composition is determined by EDX (11 kV). FEBID parameters include 5 kV acceleration voltage, 1.6 nA beam current, deposition area of 1.4 µm x 1.4 µm, 20 nm x 20 nm pitch, 1 µs dwell-time. The deposition is carried out on Au (250 nm) / Cr (8 nm) / sapphire substrates.

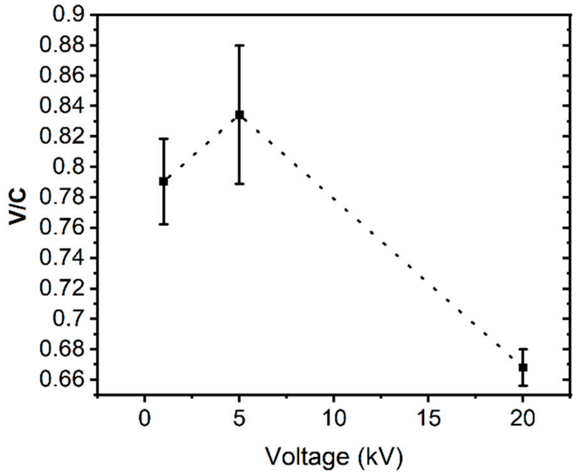

Figure S2. Evaluation of variation in V/C ratio upon changes in the acceleration voltage for FEBID using $V(CO)_6$ precursor when the acceleration voltage for FEBID deposition is varied. The deposit composition is determined by EDX (11 kV). FEBID parameters include deposition area 1.4 µm x 1.4 µm, 20 nm x 20 nm pitch, 1 µs dwell-time. The deposition is carried out on Au (250 nm) / Cr (8 nm) / sapphire substrates.

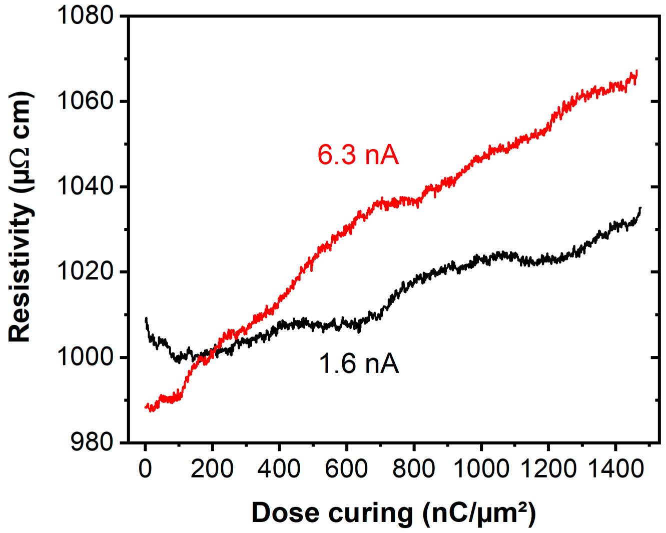

Figure S3. Changes in resistivity vs. electron dose for different deposition currents and variation in post-growth irradiation currents measured on of V-based FEBID material in two-probe configuration. Effect of dose on the materials resistivity is recorded *in situ* and illustrates only very minor variation with the electron beam current used for the curing.

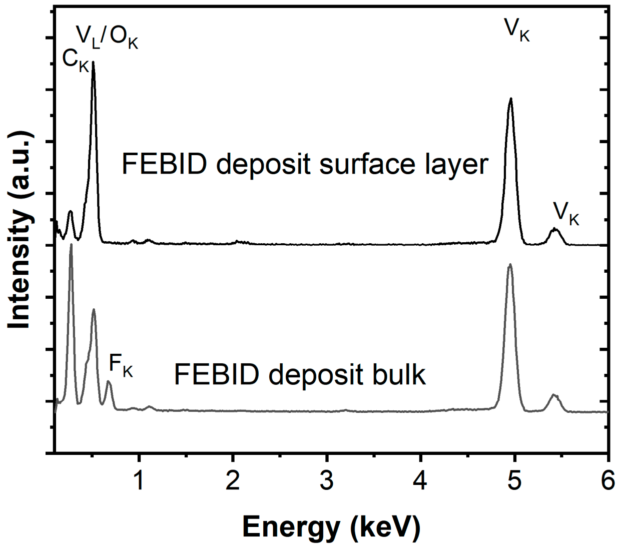

Figure S4. Comparison of TEM-EDX spectra of the FEBID deposit bulk and interface between the $VC_{1-x}O_x$ "bulk deposit" and the PtC_x protection layer deposited for TEM lamella preparation.

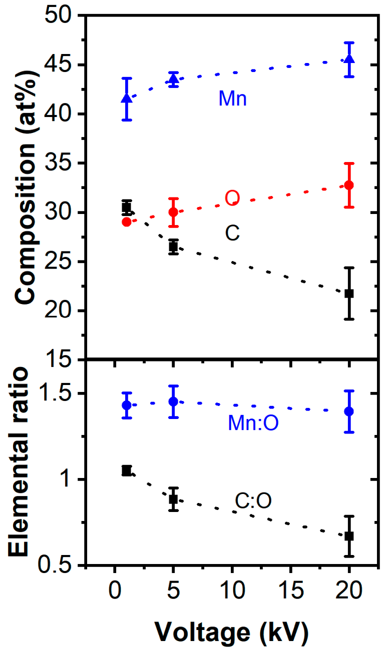

Figure S5. Evaluation of compositional variation of material derived by FEBID using $Mn_2(CO)_{10}$ precursor when the acceleration voltage for FEBID deposition is varied. The deposit composition is determined by EDX (3.5 kV). FEBID parameters include deposition area 1.4 µm x 1.4 µm, 20 nm x 20 nm pitch, 1 µs dwell-time. The deposition is carried out on Au (100 nm) / Cr (8 nm) / sapphire substrates.

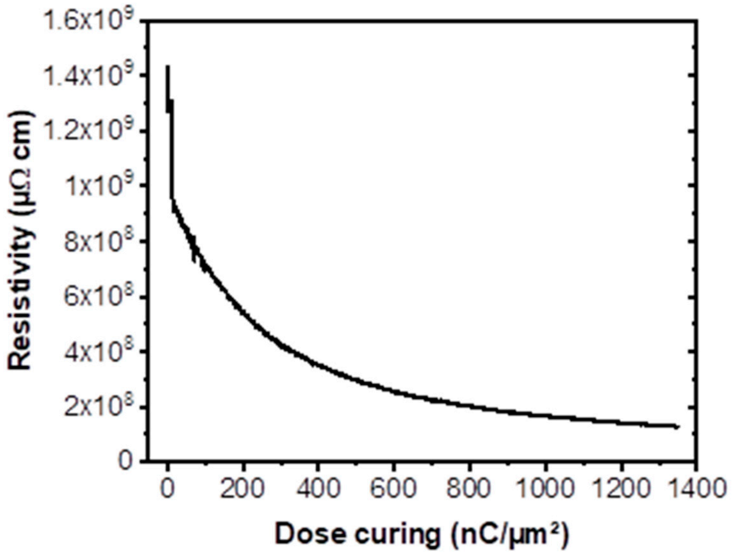

Figure S6. Changes in resistivity vs. electron dose for a Mn-based FEBID deposit in two-probe configuration. FEBID parameters include 5 kV acceleration voltage, 6.3 nA current, 20 nm x 20 nm pitch, 1 μs dwell-time. The effect of post-growth irradiation dose on the materials resistivity is recorded *in situ* and illustrates a decrease by ~one order of magnitude.
